# Association between secondary thrombocytosis and viral respiratory tract infections in children

**DOI:** 10.1038/srep22964

**Published:** 2016-03-11

**Authors:** Shou-Yan Zheng, Qiu-Yan Xiao, Xiao-Hong Xie, Yu Deng, Luo Ren, Dai-Yin Tian, Zheng-Xiu Luo, Jian Luo, Zhou Fu, Ai-Long Huang, En-Mei Liu

**Affiliations:** 1 Pediatric Research Institute, Children's Hospital of Chongqing Medical University, Ministry of Education Key Laboratory of Child Development and Disorders, China International Science and Technology Cooperation base of Child Development and Critical Disorders, Chongqing Key Laboratory of Pediatrics, Chongqing, 400014, China; 2 Department of Respiratory Medicine, Children’s Hospital of Chongqing Medical University, Chongqing, 400014, China; 3 Key Laboratory of Molecular Biology of Infectious Diseases, Ministry of Education, Chongqing Medical University, Chongqing, 400014, China

## Abstract

Secondary thrombocytosis (ST) is frequently observed in children with a variety of clinical conditions. The leading cause of ST is respiratory tract infection (RTI) in children. Nasopharyngeal aspirate samples were collected and assessed for common respiratory viruses. The relationships between virus infections and secondary thrombocytosis were analyzed retrospectively. The blood platelet count and the presence of respiratory viruses were determined for 3156 RTI patients, and 817 (25.9%) cases with platelet ≥500 × 10^9^/L were considered as the thrombocytosis group. Compared with the normal group, the detection rates of respiratory syncytial virus (RSV) and human rhinovirus (HRV) were significantly higher in the thrombocytosis group (P = 0.017 and 0.042, respectively). HRV single infection was a risk factor associated with thrombocytosis [odds ratio (OR) = 1.560, 95% confidence interval (CI) = 1.108–2.197]. Furthermore, ST was more likely to occur in younger patients who had clinical manifestations of wheezing and dyspnea and who had been diagnosed with bronchiolitis. Furthermore, the course of disease lasted longer in these patients. ST is associated with viral respiratory tract infections, especially RSV and HRV infections. HRV single infection is a risk factor associated with thrombocytosis.

An increased platelet count is common in pediatric patients, and this abundance of platelets can result in a hypercoagulable state or thrombogenesis, which is of great concern to the patients’ parents and medical staff. Thrombocytosis refers to a platelet count above the normal value and can be classified as primary or essential or as secondary or reactive. Few published studies have examined the etiology and mechanisms of thrombocytosis.

Primary thrombocytosis is a myeloproliferative disorder that is rare in children^1^. However, secondary thrombocytosis (ST) is frequently observed in children that have a variety of clinical conditions. The potential underlying causes include acute bacterial or viral infections, tissue damage, cancer and chronic inflammation, particularly during early life^2^,^3^. The most frequent cause of ST is respiratory tract infections (RTIs) in childhood; RTIs account for 60–80% of ST cases^4^,^5^,^6^. However, reports of thrombocytosis induced by viral infections are scarce, particularly in children. Previous studies have suggested that thrombocytosis may be an early marker of respiratory syncytial virus (RSV) infection, and several authors have reported that RSV-positive bronchiolitis in hospitalized young infants is associated with thrombocytosis^7^,^8^. Another study reported that thrombocytotic patients have a more severe clinical condition and suggested that the platelet count may be a useful clinical marker associated with the severity of the lower respiratory tract infection^9^. Conversely, some authors have found that platelet counts do not correlate with outcomes, disease activity, or the severity of the infections^10^.

The aim of our present study was to determine the relationship between viral respiratory tract infections and ST in hospitalized children with respiratory tract infections. Further evaluations of whether ST is related to demographic, clinical or laboratorial characteristics will be useful for clinical management of this condition.

## Materials and Methods

### Ethics statement

All experiments were approved by the Ethics Committee of the Children’s Hospital of Chongqing Medical University. The guardians of the patients signed informed consent for participation in this study and for the publication of the individual clinical details. The methods were carried out in accordance with the approved guidelines. The study was conducted in compliance with the principles of the Declaration of Helsinki.

### Study subjects and samples collection

Children with respiratory tract infections (RTIs) that were treated at the Department of Respiratory Medicine at the Children’s Hospital of Chongqing Medical University in China between June 2009 and May 2014 were enrolled in the study. In all patients, the diagnosis of RTI was based on clinical, laboratorial and radiological evidence. Nasopharyngeal aspirate (NPA) samples were collected when the patients were admitted to our department. The specimens were kept at 4 °C for a maximum of 4 h and stored at −80 °C until further processing.

### Methods of diagnosis for viruses

The viral DNA and RNA were extracted from 200-μl aliquots of the NPA samples using a QIAampMinElute Virus Spin Kit (Qiagen, Hilden, Germany). The RNA was applied as the template for cDNA synthesis using the SuperScript III First-Strand Synthesis System (Invitrogen, California, USA). The DNA and RNA extractions and cDNA products were used for subsequent testing for respiratory viruses. All of the samples were analyzed using a commercial detection kit (TaKaRa Biotechnology, Dalian, China and Applied Biosystems, California, USA), according to the manufacturer’s instructions. Multiplex nested polymerase chain reaction (PCR) was used to detect the following common respiratory viruses, as described previously^11^,^12^,^13^,^14^,^15^: RSV subtypes A and B (RSVA, RSVB); influenza virus (IFV) subtypes A, B and C (IFVA, IFVB, IFVC); human coronaviruses (HCoV); metapneumovirus (MPV); parainfluenza virus type 1 to 4 (PIV1-4); adenovirus (ADV); human bocavirus type 1 (HBoV1) and human rhinovirus (HRV) subtypes A and C (HRVA, HRVC). Real-time PCR (RT-PCR) was also used to detect HRV and HBoV1. HRV-positive samples were further amplified and sequenced to identify the HRV subtype.

### Platelet counting and thrombocytosis definition

Complete blood counts were performed in the clinical laboratory using an XE-2100 blood autoanalyzer (SYSMEX, Japan). We used clinical features and published reference standards to define normal platelet counts as being between 100 × 10^9^/L and 499 × 10^9^/L for children. Platelet counts of ≥500 × 10^9^/L were defined as thrombocytosis. Moreover, to better consider the clinical characteristics and implications of thrombocytosis, patients with platelet counts between 500–699 × 10^9^/L were diagnosed with mild thrombocytosis and those with counts of ≥700 × 10^9^/L were considered as moderate to severe thrombocytosis; platelet counts above 1000 × 10^9^/L were considered indicative of extreme thrombocytosis^16^. A retrospective review of the medical records was performed on all patients, and those who had at least one platelet count ≥500 × 10^9^/L during hospitalization were recruited into the thrombocytosis group.

### Statistical analyses

Statistical analyses were performed using the Statistical Package for the Social Sciences version 17.0 (SPSS 17.0). The categorical variables were compared using the Chi-square test or Fisher exact test, and the continuous variables were compared using Student’s t-test or the nonparametric Mann-Whitney U-test. Correlation was analyzed using logistic regression analysis. P-values < 0.05 were considered to be significant.

## Results

### Demographic data and platelet count of total patients

During the five-year study period, the blood platelet count and presence of respiratory viruses were randomly assessed in 3156 children ranging in age from 1 month to 17 years (median = 7 months). Approximately 66.5% (2100) of these children were male. Based on the clinical data, 817 (25.9%) cases with platelet counts ≥500 × 10^9^/L (median 583 × 10^9^/L) were considered as the thrombocytosis group, and 2339 (74.1%) cases with platelet counts ranging between 100 and 499 × 10^9^/L (median 335 × 10^9^/L) were considered as the normal group. Among thrombocytotic patients, 657 (80.4%) with counts between 500 and 699 × 10^9^/L were considered as having mild thrombocytosis, and 160 (19.6%) patients with counts of ≥700 × 10^9^/L were classified as having moderate to severe thrombocytosis; this last group included 10 patients with platelet counts >1000 × 10^9^/L. Thrombocytosis was presented in 25.9% (817/3156) of patients and most frequently ranged from 500 to 699 × 10^9^/L. We observed that the platelet count began to elevate on approximately the 11^th^ day of illness.

The age distribution of patients with ST was 1 month to 11 years (median 5 months); most of these patients were younger than two years of age, 772 (94.5%), and were markedly younger than patients without ST (median 9 months) (P < 0.001). Moreover, 557 (68.2%) of the thrombocytotic patients were male, but the gender distributions were not significantly different from the normal platelet patients.

### Virus detection of the normal and thrombocytosis groups

The virus detection rates of the normal and thrombocytosis groups were 77.5% (1812/2339) and 78.9% (645/817), respectively. The single viral infection rates were 44.4% (1039/2339) and 44.5% (364/817), respectively, and the co-infection rates were 33.0% (773/2339) and 34.4% (281/817), respectively. There was no significant difference in the total virus detection rate between the two groups. Among the thrombocytosis group, RSV (39.5%) was the most common virus detected, followed by HRV (22.6%) and PIV (21.8%). The detection results for each virus and their different subtypes for both groups are summarized in Table 1. Compared with the normal group, the total detection rates of RSV and HRV were both significantly higher (P = 0.001 and 0.033, respectively) in the thrombocytosis group, and ADV and IFV were lower (P = 0.001 and 0.007, respectively).

The single viral infection rates of RSV and HRV in the thrombocytosis group were both significantly higher (P = 0.017 and 0.042, respectively), and the single viral infection rates of ADV and IFV in thrombocytosis group were significantly lower compared to the normal platelet group (P < 0.001 and P = 0.004, respectively). However, the co-infection rate of viruses between two groups was not remarkably different except for RSV. The specific virus detection results are summarized in Table 2.

We further divided the thrombocytosis group into mild and moderate to severe groups and analyzed the viral infections of these subgroups. There was no marked difference of the virus detection rate between the two groups, with the exception that HBoV1 co-infections were more common in the mild thrombocytosis group (Tables 3 and 4).

We also divided the normal platelet counts group into two subgroups: (100–299) × 10^9^/L and (300–499) × 10^9^/L, then analyzed the viral infections of the subgroups. The total detection rates of RSV and PIV were both significantly higher (P = 0.002 and 0.004, respectively) in the latter group, and ADV and IFV were lower (P < 0.001 and 0.024, respectively) (Table 5).

### Clinical data in the normal and thrombocytosis groups

We compared the clinical conditions of the two groups, and the clinical manifestations of wheezing and dyspnea were significantly overrepresented in the thrombocytotic group patients compared to the normal group patients (P < 0.001 and P = 0.006, respectively). However, the occurrence of fever in the normal group was remarkable higher than the thrombocytosis group. The most common clinical diagnoses of the two groups were lower respiratory tract infections, such as pneumonia, bronchiolitis, severe pneumonia, severe bronchiolitis and bronchitis. Other conditions included upper respiratory tract infection, laryngitis, and amygdalitis. The incidence of bronchiolitis in the thrombocytosis group was significantly higher than in the normal group (P < 0.001). The days of illness before admission, length of hospitalization and course of disease in the thrombocytosis group were all longer compared with the normal group (P < 0.001). The C-reactive protein (CRP) levels of 132 (16.1%) thrombocytotic patients were above 8 mg/L. The percentage of patients with elevated CRP levels was not significantly different between two groups, but the rate of anemia in the thrombocytosis group was higher (P = 0.030). In the thrombocytosis group, sputum culture-positive results were obtained in 360 (44.0%) cases, and virus detection and sputum cultures were both positive in 284 (34.8%) cases. The typical clinical findings are summarized in Table 6.

Moreover, we also investigated patients with very high platelet counts. There were 10 patients with extreme thrombocytosis, defined as platelet counts of >1000 × 10^9^/L; the maximum count was 1504 × 10^9^/L. Of these patients, 9 were less than one year old, 4 were diagnosed with bronchiolitis, and 6 were diagnosed with pneumonia. In 7 of these cases, at least one viral infection was detected, and the primary viruses were RSVA, PIV3, and HRVA.

### Association between thrombocytosis, clinical features and viral infection from the logistic regression analysis

The age distribution, occurrence of wheezing, prevalence of bronchiolitis, course of disease and viral infection were further analyzed using logistic regression models. In general, HRV single infection was a risk factor associated with thrombocytosis (OR = 1.560, 95% CI = 1.108–2.197) after considering the effects of age, course of the disease, occurrence of wheezing and diagnosis with bronchiolitis. HRVA was especially strongly associated with thrombocytosis (OR = 1.803, 95% CI = 1.139–2.856) (Table 7).

### Complication and prognosis of thrombocytosis group

In the thrombocytosis group, no one developed thromboembolic or hemorrhagic complications. In total, 795 (97.3%) patients reached clinical recovery or improvement, 19 (2.3%) patients did not improve and 3 (0.4%) patients died of respiratory failure and septicemia.

## Discussion

ST is primarily found in pediatric patients with respiratory tract infections. In our study, ST was identified in approximately 25.9% (817) of the 3156 inpatients with RTI. ST was especially common in patients with lower respiratory tract infections and occurred most frequently in the mild thrombocytosis group (500–699 × 10^9^/L). Previous studies showed that ST occurs in 9–48% of patients with respiratory tract infections^7^,^8^,^9^. The different incidence rates may be explained by the cutoff platelet count, study population (inpatients, outpatients, or both), and median age of the enrolled subjects. Additionally, the platelet count peaked on the 11^th^ day after the onset of illness. This also supports the findings by other investigators who reported that platelet count in ST peaked during the second and third week of the illness^9^,^17^. In our cases, the highest incidence of ST occurred in patients who were under two years of age (94.5%), which is consistent with the Mastubara *et al.* study of Japanese children with thrombocytosis, more than 80% of whom were aged less than two years^18^. Because bone marrow precursor cells of younger infants are more sensitive and rapid to external stimuli such as infections.

Our findings showed that childhood ST is related to respiratory viral infections, but the degree of platelet elevation is unrelated to the species of virus. The total virus detection rate was 78.9% (645/817) in patients with ST. RSV (39.5%) was the most commonly detected virus, followed by HRV (22.6%) and PIV (21.8%). From the present data, we found that RSV single infection (especially subtype B) and HRV infection (especially subtype A) were prominent causative agents of thrombocytosis. To our knowledge, our study is the first to find that ST occurrence is associated with HRV infection. In addition, HBoV1 co-infection with other viruses in the mild thrombocytosis group is more common than in the moderate to severe thrombocytosis groups. This may be because this group of patients is frequently co-infected with RSV and HRV. According to the above results, we speculate that increased platelet counts may be indicative of a respiratory tract inflammatory reaction after RSV or HRV infection.

The mechanism by which infection can promote thrombocytosis has not yet been fully elucidated. Reports in the literature show that ST is related to increased endogenous levels of several cytokines, such as thrombopoietin (TPO), interleukin-6 (IL-6), interleukin-8 (IL-8), interleukin-1alpha (IL-1a) and tumor necrosis factor alpha (TNF-a)^19^,^20^,^21^. It has been found that during RSV infection, many infants demonstrated RSV-specific cytokine responses, such as increased levels of IL-6 and IL-8^22^. However, ST caused by HRV infection has not been described. Bronchial epithelial cells secrete a wide variety of inflammatory cytokines, including IL-1, IL-6, IL-8, and GM-CSF, and these cytokines are responsible for the development of thrombocytosis following RSV and HRV infections^23^,^24^. Furthermore, TPO concentrations usually correlate with CRP levels^25^,^26^. Among our thrombocytotic patients, 16.1% (132) had elevated CRP levels; this could indicate that CRP participates in thrombocytosis. Some authors have suggested that anemia may also play a role, and we observed a higher rate of anemia in the thrombocytosis group^5^,^7^. Another theory confirmed that the number of megakaryocytes increased and that the pulmonary capillary bed released more platelets due to stimulation by TPO after infection. We believe the specific viral agents that cause RTI induce inflammatory response and circulating cytokines that can then lead to ST and perhaps other hematological disorders.

Patients that develop ST have been more likely to also experience wheezing, dyspnea and a longer disease course. Therefore, increased platelet counts may be a clinical marker associated with the severity of RTI. Our logistic regression analysis showed that patients who had clinical manifestations of wheezing, bronchiolitis and a longer course of disease were more inclined to develop thrombocytosis. Additionally, HRV single infection was a risk factor associated with thrombocytosis. Therefore, physicians should carefully monitor these patients. However, the mechanism through which this group of patients developed thrombocytosis has not been fully determined. The percentage of patients with fever in the normal group was higher than in the thrombocytosis group; this could possibly due to the presence of bacterial infections in patients with normal platelet counts.

The great majority of our patients underwent clinical recovery or improvement. In childhood, ST rarely results in thromboembolic or hemorrhagic complications, so treatment with platelet aggregation inhibitors is not required even when the platelet count is more than 1000 × 10^9^/L. Treatment should be targeted at the primary disease rather than the platelet count^27^.

We observed that the detection rate of ADV and IFV was lower in children with ST. Generally, cytomegalovirus (CMV), Epstein-Barr virus (EBV) and Parvoviridae often lead to thrombocytopenia^28^,^29^. One explanation for this observation could be that ADV infection is more common in older children that do not frequently develop ST. Further work is needed to determine why ADV and IFV seldom cause thrombocytosis.

The limitations of our study include that the fact that this is a retrospective study rather than a cohort study, our study was performed in a single medical center, the subjects were limited to a young age group, and we had no follow-up study of platelet change. A large-scale cohort study is needed to investigate the causal relationship between viral infections and ST in children. More specific studies are needed to further elucidate the inflammatory process involved in viral infection and the specific pathophysiology of thrombocytosis.

In summary, research regarding viral respiratory tract infections and thrombocytosis is lacking, and we performed a large sample analysis that linked a specific viral agent to thrombocytosis in this clinical retrospective study. Childhood ST is related to respiratory tract infections, the occurrence rate is approximately 25.9%, and the most common viruses are RSV and HRV, but infections with ADV or IFV seldom cause thrombocytosis. HRV single infection is a risk factor associated with thrombocytosis. Younger infants that are diagnosed with bronchiolitis and have a clinical manifestation of wheezing are more likely to develop ST and undergo a longer course of disease. These observations suggest that increased platelet counts may be a retrospective marker of respiratory tract inflammatory reactions after viral infections and can indicate the severity of lower respiratory tract infections.

## Additional Information

**How to cite this article**: Zheng, S.-Y. *et al.* Association between secondary thrombocytosis and viral respiratory tract infections in children. *Sci. Rep.* **6**, 22964; doi: 10.1038/srep22964 (2016).

## Figures and Tables

**Table 1:** Comparison of each virus total detection rate between the normal and thrombocytosis groups.

Virus	Normal group (n = 2339) (%)	Thrombocytosis group (n = 817) (%)	*P* value
**RSV**	770 (32.9)	323 (39.5)	0.001
RSVA	455 (19.5)	176 (21.5)	0.204
RSVB	304 (13.0)	140 (17.1)	0.003
**HRV**	448 (19.2)	185 (22.6)	0.033
HRVA	199 (8.5)	97 (11.9)	0.005
HRVC	245 (10.5)	79 (9.7)	0.508
**ADV**	183 (7.8)	36 (4.4)	0.001
**PIV**	487 (20.8)	178 (21.8)	0.571
PIV1	95 (4.1)	23 (2.8)	0.105
PIV2	20 (0.9)	2 (0.2)	0.071
PIV3	299 (12.8)	124 (15.2)	0.084
PIV4	28 (1.2)	7 (0.9)	0.422
**IFV**	435 (18.6)	118 (14.4)	0.007
IFVA	387 (16.5)	105 (12.8)	0.012
IFVB	38 (1.6)	8 (1.0)	0.185
IFVC	9 (0.4)	3 (0.4)	1.000
**HBoV1**	353 (15.1)	108 (13.2)	0.188
**HCoV**	48 (2.1)	20 (2.4)	0.505
**MPV**	72 (3.1)	22 (2.7)	0.573

RSV: respiratory syncytial virus; RSVA, RSVB: RSV subtypes A and B; HRV: human rhinovirus; HRVA, HRVC: HRV subtypes A and C; ADV: adenovirus; PIV: parainfluenza virus; PIV1- 4: PIV types 1 to 4; IFV: influenza virus; IFVA, IFVB, IFVC: IFV subtypes A, B and C; HBoV1: human bocavirus type 1; HCoV: human coronaviruses; MPV: metapneumovirus.

**Table 2:** Comparison of specific virus detection rates between the normal and thrombocytosis groups.

Virus	Single virus infection			>Co-infection		
	Normal group (n = 1039) (%)	Thrombocytosis group (n = 364) (%)	*P* value	Normal group (n = 773) (%)	Thrombocytosis group (n = 281) (%)	*P* value
**RSV**	353 (34.0)	149 (40.9)	0.017	412 (53.3)	173 (61.6)	0.017
RSVA	217 (20.9)	83 (22.8)	0.443	238 (30.8)	93 (33.1)	0.476
RSVB	136 (13.1)	66 (18.1)	0.018	163 (21.1)	73 (26.0)	0.092
**HRV**	140 (13.5)	65 (17.9)	0.042	308 (39.8)	120 (42.7)	0.403
HRVA	65 (6.3)	35 (9.6)	0.032	134 (17.3)	62 (22.1)	0.081
HRVC	74 (7.1)	27 (7.4)	0.851	171 (22.1)	52 (18.5)	0.204
**ADV**	89 (8.6)	10 (2.7)	<0.001	94 (12.2)	26 (9.3)	0.189
**PIV**	169 (16.3)	66 (18.1)	0.412	317 (41.0)	111 (39.5)	0.660
PIV1	31 (3.0)	9 (2.5)	0.614	63 (8.2)	14 (5.0)	0.081
PIV2	9 (0.9)	1 (0.3)	0.428	13 (1.7)	1 (0.4)	0.174
PIV3	117 (11.3)	53 (14.6)	0.097	182 (23.5)	71 (25.3)	0.563
PIV4	12 (1.2)	3 (0.8)	0.817	16 (2.1)	4 (1.4)	0.496
**IFV**	159 (15.3)	34 (9.2)	0.004	276 (35.7)	84 (29.9)	0.079
IFVA	142 (13.7)	30 (8.2)	0.007	245 (31.7)	75 (26.7)	0.118
IFVB	15 (1.4)	2 (0.5)	0.288	23 (3.0)	6 (2.1)	0.461
IFVC	2 (0.2)	2 (0.5)	0.597	7 (0.9)	1 (0.4)	0.612
**HBoV1**	93 (9.0)	30 (8.2)	0.681	260 (33.6)	78 (27.8)	0.071
**HCoV**	14 (1.4)	2 (0.5)	0.344	34 (4.4)	18 (6.4)	0.183
**MPV**	26 (2.5)	8 (2.2)	0.745	46 (6.0)	14 (5.0)	0.548

**Table 3:** Comparison of each virus total detection rate between the mild and moderate to severe thrombocytosis groups.

Virus	Mild thrombocytosis group (n = 657) (%)	Moderate to severe thrombocytosis group (n = 160) (%)	*P* value
**RSV**	252 (38.4)	71 (44.4)	0.163
RSVA	137 (20.9)	39 (24.4)	0.326
RSVB	108 (16.4)	32 (20.0)	0.284
**HRV**	155 (23.6)	30 (18.8)	0.192
HRVA	79 (12.0)	18 (11.3)	0.791
HRVC	69 (10.5)	10 (6.3)	0.163
**ADV**	28 (4.3)	8 (5.0)	0.680
**PIV**	139 (21.2)	38 (23.8)	0.496
PIV1	18 (2.7)	5 (3.1)	0.999
PIV2	2 (0.3)	0	—
PIV3	97 (14.8)	27 (16.9)	0.505
PIV4	5 (0.8)	2 (1.3)	0.900
**IFV**	93 (14.2)	25 (15.6)	0.635
IFVA	82 (12.5)	23 (14.4)	0.521
IFVB	6 (0.9)	2 (1.3)	1.000
IFVC	3 (0.5)	0	—
**HBoV1**	95 (14.5)	13 (8.1)	0.034
**HCoV**	19 (2.9)	1 (0.6)	0.169
**MPV**	14 (2.1)	8 (5.0)	0.082

**Table 4:** Comparison of specific virus detection rate between the mild and moderate to severe thrombocytosis groups.

Virus	Single virus infection			Co-infection		
	Mild thrombocytosis group (n = 283) (%)	Moderate to severe thrombocytosis group (n = 81) (%)	*P* value	Mild thrombocytosis group (n = 229) (%)	Moderate to severe thrombocytosis group (n = 52) (%)	*P*value
RSV	114 (40.3)	35 (43.2)	0.637	138 (60.3)	35 (67.3)	0.346
HRV	51 (18.0)	14 (17.3)	0.879	104 (45.4)	16 (30.8)	0.054
PIV	50 (17.7)	16 (19.7)	0.668	89 (38.9)	22 (42.3)	0.647
ADV	8 (2.8)	2 (2.5)	1.000	20 (8.7)	6 (11.5)	0.715
IFV	29 (10.2)	5 (6.2)	0.267	64 (27.9)	20 (38.4)	0.135
HBoV1	24 (8.5)	6 (7.4)	0.757	71 (31.0)	7 (13.5)	0.011
HCoV	2 (0.7)	0	—	17 (7.4)	1 (1.9)	0.251
MPV	5 (1.8)	3 (3.7)	0.536	9 (3.9)	5 (9.6)	0.178

**Table 5:** Comparison of each virus total detection rate between two normal platelet counts subgroups.

Virus	Platelet counts (100–299) × 10^9^/L (n = 910) (%)	Platelet counts (300–499) × 10^9^/L (n = 1429) (%)	*P* value
**RSV**	266 (29.2)	504 (35.3)	0.002
RSVA	160 (17.6)	295 (20.6)	0.068
RSVB	101 (11.1)	203 (14.2)	0.029
**HRV**	161 (17.7)	287 (20.1)	0.152
HRVA	66 (7.3)	133 (9.3)	0.083
HRVC	95 (10.4)	150 (10.5)	0.965
**ADV**	108 (11.9)	75 (5.2)	<0.001
**PIV**	162 (17.8)	325 (22.7)	0.004
PIV1	39 (4.3)	56 (3.9)	0.661
PIV2	6 (0.7)	14 (1.0)	0.412
PIV3	91 (10.0)	208 (14.6)	0.001
PIV4	11 (1.2)	17 (1.2)	0.967
**IFV**	190 (20.9)	245 (17.1)	0.024
IFVA	163 (17.9)	224 (15.7)	0.156
IFVB	23 (2.5)	15 (1.0)	0.006
IFVC	4 (0.4)	5 (0.3)	1.000
**HBoV1**	146 (16.0)	207 (14.5)	0.305
**HCoV**	16 (1.8)	32 (2.2)	0.424
**MPV**	29 (3.2)	43 (3.0)	0.808

**Table 6:** Comparison of clinical data between the normal and thrombocytosis groups.

Variable	Normal group (n = 2339)	Thrombocytosis group (n = 817)	*P* value
Wheezing (n, %)	931 (39.8%)	455 (55.6%)	<0.001
Dyspnea (n, %)	413 (17.7%)	180 (22.0%)	0.006
Fever (n, %)	1288 (55.1%)	345 (42.2%)	<0.001
Upper respiratory tract infection (n, %)	89 (3.8%)	9 (1.1%)	–
Lower respiratory tract infection (n, %)	2250 (96.2%)	808 (98.9%)	–
Pneumonia (n, %)	1766 (75.5%)	591 (72.3%)	0.073
Severe pneumonia (n, %)	284 (12.1%)	105 (12.9%)	0.595
Bronchiolitis (n, %)	328 (14.0%)	186 (22.7%)	<0.001
Severe bronchiolitis (n, %)	73 (3.1%)	34 (4.2%)	0.159
Days of illness before admission (day)	8 (0–365)	10 (0–60)	<0.001
Length of hospitalization (day)	6 (1–87)	7 (1–87)	<0.001
Course of disease (day)	15 (1–378)	18 (3–94)	<0.001
Percentage of CRP level increased (n, %)*	416 (17.8)	132 (16.1)	0.284
Anemia (n, %)	163 (7.0)	76 (9.3)	0.030

*CRP: C-reactive protein; CRP level above 8 mg/L were defined as increased.

**Table 7:** Association between thrombocytosis, clinical features and viral infection from the logistic regression analysis.

Virus	Age		Course of disease		Wheezing		Bronchiolitis		Single virus infection	
	OR (95% CI)	*P* value	OR (95% CI)	*P* value	OR (95% CI)	*P* value	>OR (95% CI)	*P* value	OR (95% CI)	*P* value
RSV infected	0.964 (0.952,0.976)	<0.001	1.018 (1.008,1.028)	<0.001	1.468 (1.131,1.905)	0.004	1.647 (1.189,2.282)	0.003	0.923 (0.705,1.209)	0.563
HRV infected	0.964 (0.952,0.976)	<0.001	1.017 (1.007,1.028)	0.001	1.461 (1.127,1.894)	0.004	1.661 (1.209,2.284)	0.002	1.560 (1.108,2.197)	0.011
HRVA infected	0.964 (0.952,0.976)	<0.001	1.017 (1.007,1.028)	0.001	1.469 (1.133,1.905)	0.004	1.635 (1.190,2.246)	0.002	1.803 (1.139,2.856)	0.012

OR, odds ratio; 95% CI, 95% confidence intervals.
